# Gene Expression in Class 2 Integrons Is SOS-Independent and Involves Two Pc Promoters

**DOI:** 10.3389/fmicb.2017.01499

**Published:** 2017-08-15

**Authors:** Thomas Jové, Sandra Da Re, Aurore Tabesse, Amy Gassama-Sow, Marie-Cécile Ploy

**Affiliations:** ^1^INSERM, CHU Limoges, UMR 1092, Université Limoges Limoges, France; ^2^Unité de Bactériologie Expérimentale, Institut Pasteur de Dakar Dakar, Senegal

**Keywords:** integrons, SOS response, promoter, antibiotic resistance, regulation

## Abstract

Integrons are powerful bacterial genetic elements that permit the expression and dissemination of antibiotic-resistance gene cassettes. They contain a promoter Pc that allows the expression of gene cassettes captured through site-specific recombination catalyzed by IntI, the integron-encoded integrase. Class 1 and 2 integrons are found in both clinical and environmental settings. The regulation of *intI* and of Pc promoters has been extensively studied in class 1 integrons and the regulatory role of the SOS response on *intI* expression has been shown. Here we investigated class 2 integrons. We characterized the P*intI2* promoter and showed that *intI2* expression is not regulated via the SOS response. We also showed that, unlike class 1 integrons, class 2 integrons possess not one but two active Pc promoters that are located within the *attI2* region that seem to contribute equally to gene cassette expression. Class 2 integrons mostly encode an inactive truncated integrase, but the rare class 2 integrons that encode an active integrase are associated with less efficient Pc2 promoter variants. We propose an evolutionary model for class 2 integrons in which the absence of repression of the integrase gene expression led to mutations resulting in either inactive integrase or Pc variants of weaker activity, thereby reducing the potential fitness cost of these integrons.

## Introduction

Integrons are widely used by Gram-negative bacteria to resist antibiotics. These DNA elements can acquire, exchange and express promoterless coding sequences embedded within gene cassettes (Escudero et al., 2015). The integron functional platform is composed of a gene (*intI*) that encodes a site-specific recombinase (IntI); a recombination site (*attI*); and a functional promoter (Pc), divergently oriented to the integrase gene, that allows the expression of gene cassettes (Stokes and Hall, 1989). IntI catalyzes recombination events that lead either to the incorporation of gene cassettes within the integron, or to their excision. Several integron classes can be discriminated on the basis of their IntI sequences (Collis et al., 2002). In clinical settings, five integron classes involved in the expression and dissemination of antibiotic-resistance gene cassettes have been described. Class 1 integrons prevail in most epidemiological studies in human and animals, followed by class 2 integrons (Gillings, 2014).

**FIGURE 1:**
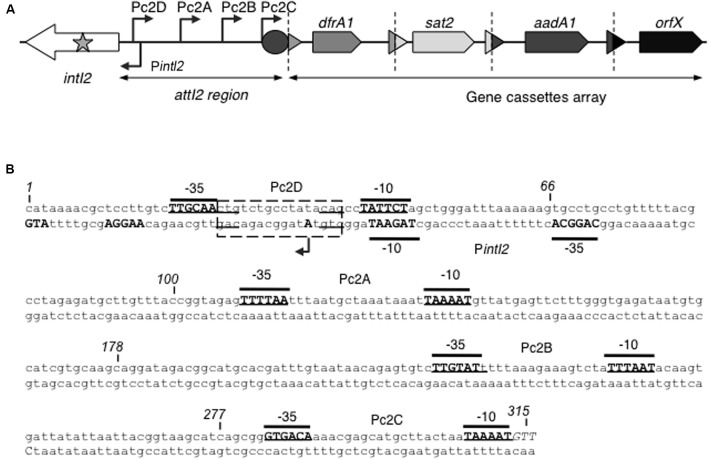
Class 2 integrons. **(A)** General structure of class 2 integrons: Arrows indicate the coding sequences with the gene name above, triangles and circles are *attC* and *attI* recombination sites, respectively. The *attI2* region and gene cassette array are indicated. Dotted vertical bars separate each gene cassette. The putative promoters are shown as broken arrows with their names indicated. *orfX* is a pseudocassette whose *attC* site is incomplete. The star symbolizes the nonsense mutation that disrupts most *intI2* genes. **(B)** Nucleotide sequence of the *attI2* region. The -35 and -10 elements of the putative promoters are written in bold uppercase, and their names are indicated. The *intI2* START codon and its putative RBS are written in bold uppercase on the bottom strand. The transcriptional (+1) mapped for P*intI2* is indicated by a broken arrow and bold uppercase. The putative LexA box is represented as a dotted rectangle. The position of several nucleotides is numbered (italics).

In class 1 integrons, hundreds of distinct gene cassette arrays have been described (Moura et al., 2009). Class 2 integrons are associated with transposons related to Tn*7* (Cambray et al., 2010) and usually carry three resistance-encoding cassettes designated *dfrA1*, *sat2* and *aadA1* (encoding resistance to trimethoprim, streptothricin and streptomycin/spectinomycin, respectively), followed by a pseudocassette of unknown function (*orfX*, also known as *ybeA*) (**Figure 1A**) (Hall and Stokes, 1993). Variations in this gene cassette array have rarely been described (Biskri and Mazel, 2003; Ahmed et al., 2005; Ramírez et al., 2005, 2010; Barlow and Gobius, 2006; Dubois et al., 2007; Gassama Sow et al., 2008; Márquez et al., 2008). This low diversity of the gene cassette array is thought to be due to disruption of the integrase gene *intI2* by an internal ochre STOP codon (TAA) at position 179, yielding an inactive 178-aa polypeptide (Hansson et al., 2002) (**Figure 1A**). Few class 2 integrons with a non-disrupted *intI2* gene encoding a 325-aa full-length functional integrase have been described (Barlow and Gobius, 2006; Márquez et al., 2008; Rodríguez-Minguela et al., 2009; Wei et al., 2014). A putative promoter for *intI2*, hereafter named P*intI2*, has been proposed in the annotation of the R483 plasmid (GenBank accession number L10818, **Figure 1B**). This P*intI2* promoter overlaps a putative LexA repressor operator conserved in many integron classes (Cambray et al., 2011), suggesting that *intI2* could be under the control of the SOS response, like the *intI1* gene of class 1 integrons and *intIA* of the chromosomal integron of *Vibrio cholerae* (Guerin et al., 2009).

Gene cassette expression depends on the Pc promoter which, in class 1 and 3 integrons, is located within the *intI* coding sequence (Collis and Hall, 1995; Collis et al., 2002). In class 2 integrons, however, no Pc promoter sequence has been found within the *intI2* gene, and part of the *attI2* region seems sufficient for gene cassette expression (Hansson et al., 2002) (**Figure 1A**). Four putative Pc promoters, hereafter renamed Pc2A to Pc2D, have been proposed in the sequence between the start codon of the *intI2* gene and the first gene cassette (**Figure 1**) (Simonsen et al., 1983; Hansson et al., 2002 and Genbank accession number AM261760). A transcriptional start site consistent with the Pc2A promoter was recently mapped (da Fonseca et al., 2011). However, none of these potential Pc2 promoters has been experimentally characterized.

The aim of this study was to examine the expression of both the integrase and the gene cassettes of class 2 integrons, and to evaluate the role of the SOS response in class 2 integrons integrase expression. We mapped the P*intI2* promoter and found that despite the presence of a potential LexA binding site, P*intI2* is not under control of the SOS response. We also found that two promoters, Pc2A and Pc2B, seem to contribute equally to the expression of gene cassettes in class 2 integrons.

## Materials and Methods

### Bacteria and Growth Conditions

The bacterial strains and plasmids used in this study are listed in **Table 1**. Cells were grown at 37°C in lysogeny broth (LB) supplemented when necessary with kanamycin (Km, 25 μg/ml).

**Table 1:** Strains and plasmids used in this study.

Strains/plasmids	Genotype or description	Source or reference
***E. coli* strains**		
DH5aaa	(F^-^) *endA1 supE44 thi-1 recA1 relA1 gyrA96 deoR nupG*ϕ80 Δ*lacZ*Δ*M15*Δ (*lacZYA- argF*)U169, *hsdR*17(rK^-^ mK^+^), bbb–	Laboratory collection
MG1656	MG1655*lac*-	Espéli et al., 2001
MG1656Δ*lexA*	MG1656Δ*lexA*Δ*sfiA*	Guerin et al., 2009
MG1656Δ*recA*	MG1656Δ*recA*	Da Re et al., 2009
***S. sonnei* strain**		
isolate Dak 0898/12-14	Isolate carrying a class 2 integron	Gassama Sow et al., 2008
**Plasmids**		
pAT674	6.5-kb *Bam*HI fragment from In40 class 1 integron cloned into pBGS18	Ploy et al., 1998
pSU38Δtot*lacZ*	Vector carrying *lacZ* coding sequence with no translation initiation region nor promoter.	Jové et al., 2010
pPintI2-1	Whole *attI2* region amplified with primers 1 and 2 from isolate Dak 0898-14 cloned into pSU38Δtot*lacZ* to obtain P*intI2*-*lacZ* fusion.	This study
pPintI2-2	P*intI2* promoter amplified with primers 2 and 3 from isolate Dak 0898-14 cloned into pSU38Δtot*lacZ*.	This study
pPintI2-3	-10 sequence of P*intI2* mutated (TAGAAT mutated into cgGAcg) in pPintI2 with primers 4 and 5.	This study
pPc2-1	*attI2* region + beginning of *dfrA1* amplified with primers 10 and 12 from isolate Dak 0898-14 cloned into pSU38Δtot*lacZ* to obtain Pc-*lacZ* translational fusion.	This study
pPc2-2	pPc2-1 plus part of *intI2* (PCR product amplified with primers 12 and 13)	This study
pPc2-3	pPc2-1 deleted of Pc2D (PCR product amplified with primers 12 and 14)	This study
pPc2-4	pPc2-1 deleted of Pc2D and A (PCR product amplified with primers 12 and 15)	This study
pPc2-5	pPc2-1 deleted of Pc2D, A and B (PCR product amplified with primers 12 and 16)	This study
pPc2-5^∗^	pPc2-4 with the -10 sequence of Pc2C mutated (TAAAAT mutated into cgAAAT) with primers 24 and 25	This study
pPc2-6	pPc2-1 deleted of Pc2D, A, B and C (PCR product amplified with primers 12 and 17)	This study
pPc2-7	pPc2-1 mutated with primers 20 and 21 to inactivate Pc2A	This study
pPc2-8	pPc2-1 mutated with primers 22 and 23 to inactivate Pc2B	This study
pPc2-9	pPc2-1 mutated with primers 20/21 and 22/23 to concomitantly inactivate Pc2A and Pc2B	This study
pPc2-10	pPc2-1 mutated with primers 24 and 25 to inactivate Pc2C	This study
pPc2-11	pPc2-1 mutated with primers 26 and 27 to create the variant of Pc2A	This study
pPc2-12	pPc2-1 mutated with primers 28 and 29 to create the variant of Pc2B	This study
pPc2-13	pPc2-1 mutated with primers 26/27 and 28/20 to concomitantly introduce the variants of both Pc2A and Pc2B	This study
pPc2A	Pc2A amplified with primers 14 and 18 from isolate Dak 0898-14 cloned into pSU38Δtot*lacZ*	This study
pPc2A^∗^	-10 sequence of Pc2A mutated (TAAAAT into cgAgcg) in pPc2A mutated with primers 20 and 21.	This study
pPc2B	Pc2B amplified with primers 15 and 19 from isolate Dak 0898-14 cloned into pSU38Δtot*lacZ*.	This study
pPc2B^∗^	-10 sequence of Pc2B mutated (TTTAAT mutated into TTcgAT) in pPc2B mutated with primers 22 and 23	This study
pPc2A-V2	pPc2A mutated with primers 26 and 27 to create the second variant of Pc2A	This study
pPc2B-V2	pPc2B mutated with primers 28 and 29 to create the second variant of Pc2B	This study

### *lacZ* Transcriptional Fusions

Plasmids pPc2, pPintI2 and their derivatives were constructed by cloning, into the EcoRI–BamHI sites of pSU38Δtot*lacZ* (**Table 1**), a PCR product amplified either from genomic DNA of the *Shigella sonnei* isolate Dak 0898/12-14 carrying a class 2 integron previously described in the lab (Gassama Sow et al., 2008) or by assembly PCR (see below). All cloned fragments were verified by sequencing. All oligonucleotides were purchased from Sigma–Aldrich and are listed in Supplementary Table S1. Each *lacZ* fusion plasmid was transformed into *Escherichia coli* strain MG1656 (**Table 1**).

### Assembly PCR

Assembly PCR was used to mutate the Pc2 or P*intI2* promoter, using overlapping primers that contained the desired mutation, and two external primers, as previously described (Jové et al., 2010).

### 5′Rapid Amplification cDNA Ends (5′RACE)

Total RNA from *E. coli* MG1656/pPintI2-1 (**Table 1**) was extracted and cDNA specific to the *lacZ* gene was synthetized by a reverse transcriptase (TaKaRa) using primers 30, 31, and 32 (Supplementary Table S1). After purification, cDNA was used as template for the 5′RACE experiment in accordance with the manufacturer’s recommendations (5′RACE System for Rapid Amplification of cDNA Ends, Invitrogen), and using the TaKaRa Ex Taq^TM^ DNA polymerase (TaKaRa Biotechnology). The purified PCR product was cloned in the pGEM^®^-T Easy vector (Promega) in *E. coli* DH5aaa and nine clones were sequenced.

### β-Galactosidase Assays

Assays were performed with 0.5-ml aliquots of exponential-phase cultures (OD_600_ = 0.6–0.8) as described by Miller (1992) except that the incubation temperature was 37°C. Experiments were done at least five times for each strain. Treatment with mitomycin C was carried out as previously described (Guerin et al., 2009). One-way ANOVA statistical followed by *post hoc* Tukey HSD statistics tests were used to determine whether variation in expression levels were significant (*p*-values < 0.01).

### Electrophoresis Mobility Shift Assays (EMSA)

Over-expression and purification of the LexA protein was performed as previously described (Da Re et al., 2009). The EMSA probes were obtained by PCR using oligonucleotides 8 and 9 (probe PintI1, 270bp, Supplementary Table S1) or 10 and 11 (probe PintI2, 233bp, Supplementary Table S1) amplified from pAT674 or genomic DNA of isolate Dak 0898/12-14, respectively. They were end-labeled with [γ^32^P]ATP (Amersham, Saclay, France) using T4 DNA polynucleotide kinase (Promega, Charbonnières, France). The EMSA experiments were performed as previously described (Da Re et al., 2009) using various amounts of purified LexA, 40 ng of the radiolabelled DNA probe PintI1 or PintI2 in the binding mixtures, and 630 ng of unlabelled probe for competition experiments (around 15.75-fold excess).

## Results

### Mapping of the P*intI2* Promoter

**FIGURE 2:**
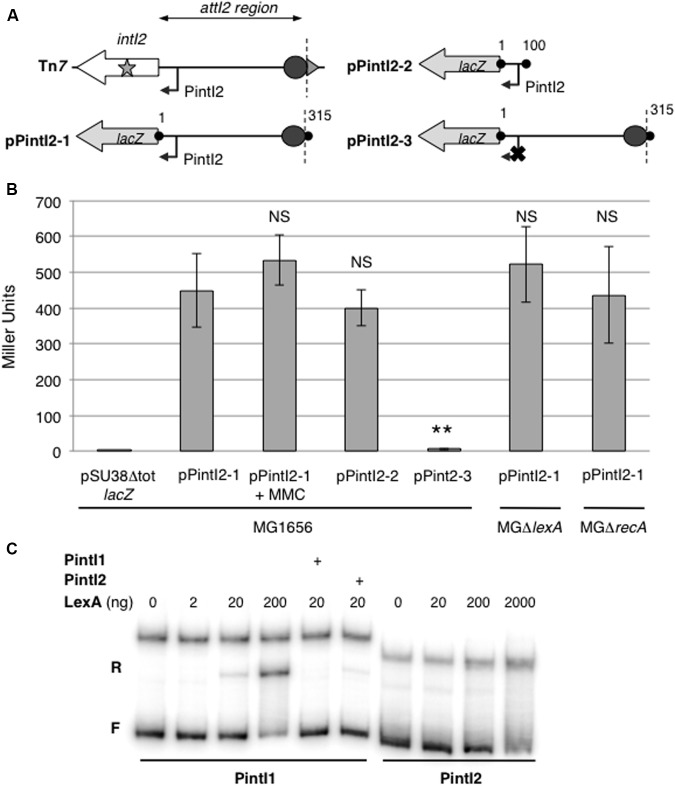
Characterization and regulation of the P*intI2* promoter. **(A)** Schematic representation of the class 2 integron carried on Tn*7* and PintI2-*lacZ* translational fusions with the entire *attI2* site or P*intI2* promoter only carried on plasmids pPintI2-1, pPintI2-2, and pPintI2-3 (pPintI2-3 carrying an inactive PintI2). Boundaries of the cloned fragment are indicated by black dots and numbered according to the *attI2* region as shown in **Figure 1B**. The broken arrow represents the putative PintI2 promoter. The boundaries of the *attI2* region (nt4-nt314) are marked by vertical dotted lines. The inactivating mutation of the -10 sequence of PintI2 is indicated by a cross. **(B)** The strength of the P*intI2* promoter was measured in β-galactosidase assays with various P*intI2*-*lacZ* translational fusions in the wild-type strain MG1656 and in its Δ*lexA* and Δ*recA* derivatives. MMC: One-hour incubation with 1.6 mg/mL mitomycin C. At least five independent assays were performed for each plasmid. Error bars indicate standard deviation. Student’s *t*-test was used for comparisons with MG1656/pPintI2-1: ^∗∗^*p* < 0.01; NS *p* > 0.05. **(C)** Electrophoresis mobility shift assay with the native P*intI1* and P*intI2* promoters, in the presence or absence of purified LexA protein (amounts are indicated in nanograms). The + sign indicates competition experiments performed with an excess of cold PintI1 or PintI2 as indicated on the side of the graph. F, free DNA; R, specific retarded complex.

To precisely identify the P*intI2* promoter, we used the 5′RACE technique (Frohman, 1993). The P*intI2* transcription start site (TSS) was mapped at position -33 upstream from the IntI2 START codon, in agreement with previously inferred potential -35 and -10 elements (respectively, CAGGCA and TAGAAT, separated by 17 bp; GenBank accession number L10818; **Figure 1B**). Downstream of P*intI2* lies a well-conserved putative translation initiation region (TIR; AAGGA-N7-ATG, see **Figure 1B**) compared to the bacterial TIR consensus sequence in *E. coli* (TAAGGA-N5/7-ATG) (Kozak, 2005). To experimentally validate P*intI2*, we tested its ability to drive the expression of the *lacZ* reporter gene by measuring β-galactosidase activity from two transcriptional fusions expressed in *E. coli* strain MG1656 and carried on plasmids pPintI2-1 and pPintI2-2 (**Table 1**). Plasmid pPintI2-1 includes the whole *attI2* region fused to *lacZ*, while pPintI2-2 is restricted to the region corresponding to the putative P*intI2* promoter (**Figure 2A**). Both constructs include the native TIR of *intI2*. We found similar levels of β-galactosidase activity with pPintI2-1 and pPintI2-2 (**Figure 2B**). This activity was abolished by mutation of the most highly conserved bases of the PintI2-10 element, with respect to the σ^70^ promoter consensus (pPintI2-3; **Table 1** and **Figures 2A,B**). These results confirmed the presence of a single functional P*intI2* promoter, CAGGCA-N17-TAGAAT.

### Expression of *intI2* Is Not Controlled by the SOS Response

Expression of the *intI* gene from P*intI* promoters in class 1 and *V. cholerae* chromosomal integrons is controlled by the LexA-mediated SOS response (Guerin et al., 2009). In class 2 integrons, a potential LexA binding site overlaps with the P*intI2* promoter (**Figure 1B**). To determine whether LexA regulates the expression of *intI2*, we measured β-galactosidase activity from pPintI2-1 in a *lexA*-deleted MG1656 derivative (**Table 1**). Surprisingly, *lexA* deletion had no significant effect on β-galactosidase activity, nor did *recA* deletion or treatment with mitomycin C (induction of the SOS response) (**Figure 2C**).

We performed EMSA with purified LexA protein and a PCR product encompassing P*intI2*. We showed that, unlike the promoter of class 1 integron integrase P*intI1* (Guerin et al., 2009), there was no specific gel shift with P*intI2*, meaning that LexA did not bind the putative LexA-binding site identified in the *attI2* region (**Figure 2C**).

Together, these results strongly indicate that, despite the presence of a good canonical binding site for LexA protein within P*intI2*, this protein does not repress *intI2* transcription, and the SOS response does not control *intI2* expression.

### Several Active Pc Promoters in the *attI2* Region

**FIGURE 3:**
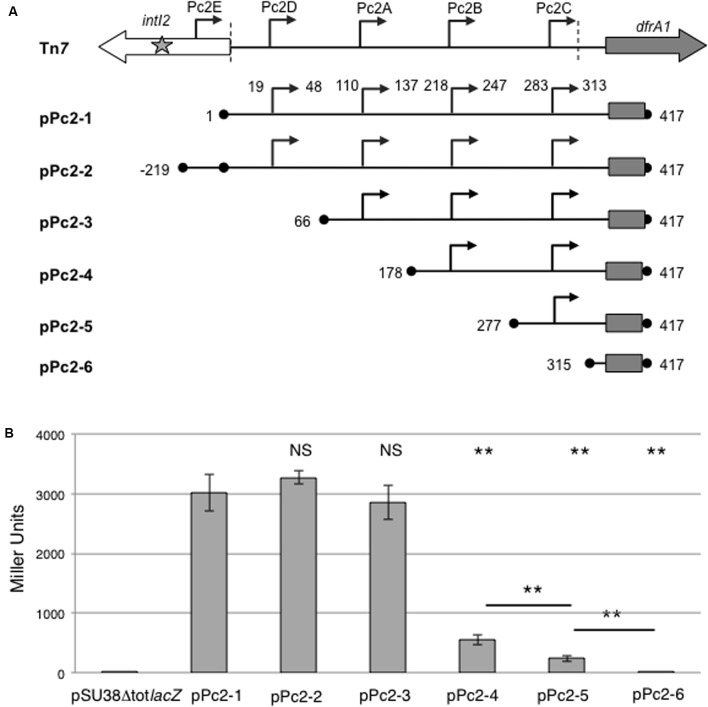
Activity of putative class 2 integron Pc promoters. **(A)** Schematic representation of the class 2 integron carried on Tn*7* and Pc-*lacZ* translational fusions with the entire or partially deleted *attI2* site carried on plasmids pPc2-1–pPc2-6. Boundaries of the cloned fragment are indicated by black dots and numbered according to its position in the pPc2-1 fragment (417 bp long). The cloned portion of the *dfrA1* coding sequence is indicated by a rectangle. Broken arrows represent each putative Pc2 promoter. The boundaries of the *attI2* region (nt4-nt314) are marked by vertical dotted lines. **(B)** The contribution of each putative Pc2 promoter (Pc2A–Pc2E) was estimated by measuring β-galactosidase activity from each plasmid. pSU38Δtot*lacZ* is the empty control plasmid. At least five independent assays were performed for each construct. Error bars indicate the standard deviation. *P*-values (ANOVA and HSD Tukey’s test) for comparison with pPc2-1 (on the top) and between constructs are indicated: ^∗∗^*p* < 0.01, NS *p* > 0.05.

Four potential Pc2 promoters have previously been inferred (named here Pc2A to Pc2D), all located within the *attI2* region. We noticed the presence of another potential promoter located within the *intI2* encoding sequence (here named Pc2E), that displays a TGN-10 motif known to increase the strength of promoters (Mitchell et al., 2003) (TGGCTA-N13TGN-TAAGCT, 165-bp away from *intI2* START codon; **Figure 3A**).

We studied the ability of these candidate Pc promoters to drive expression of the *lacZ* reporter gene, by successively deleting the putative promoters (pPc2-1 to pPc2-6; **Figure 3A** and **Table 1**). The strong activity measured from pPc2-1 (entire *attI2* region) argued for the presence of at least one functional promoter in *attI2* (**Figure 3B**). When we added Pc2E (pPc2-2) or deleted Pc2D (pPc2-3), β-galactosidase activity was similar to that obtained with pPc2-1, showing that neither Pc2D nor Pc2E significantly contributes to gene cassette transcription in class 2 integrons (**Figure 3B**). We assumed that the copy number was stable regardless the nature of the cloned fragment. Nevertheless, we cannot exclude that the small differences of promoter strength observed with Pc2-1, Pc2-2, and Pc2-3 could be explained by small variations of copy number. Nevertheless, these small differences of β-galactosidase activities were non-significant (*p* > 0.05, **Figure 3B**).

On the contrary, deletion of Pc2A (pPc2-4) reduced β-galactosidase activity by 80%, and concomitant deletion of Pc2B (pPc2-5) halved the remaining activity, leaving a residual activity of less than 10%, which could be attributed to Pc2C (**Figure 3B**). As expected, when none of the Pc2 promoters remained in the construct (pPc2-6), no β-galactosidase activity was detected (**Figure 3B**).

These results suggested that three of the four potential promoters located in the *attI2* region, namely Pc2A, Pc2B, and Pc2C, may contribute to gene cassette expression, and that Pc2A would be the major actor.

### Promoters Pc2A and Pc2B Contribute to Gene Cassette Expression

**FIGURE 4:**
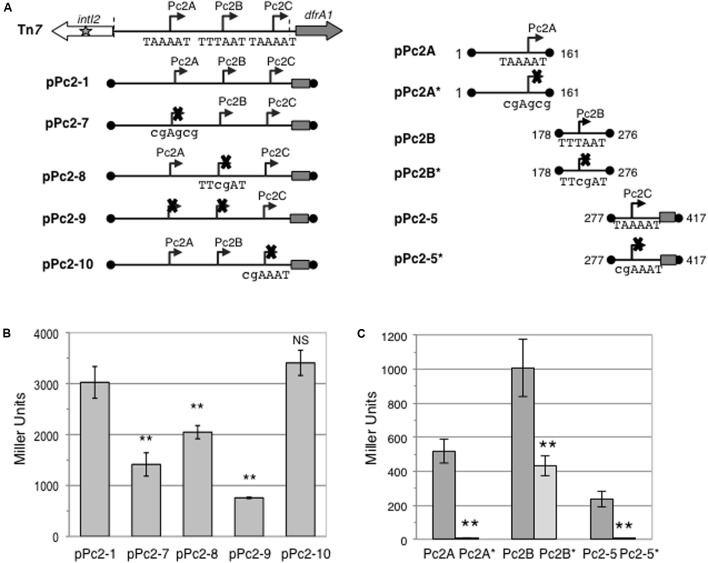
Specific contributions of the Pc2A, Pc2B and Pc2C promoters. **(A)** Schematic representation of the class 2 integron carried on Tn*7*, Pc-*lacZ* translational fusions with the entire or partially deleted *attI2* site carrying functional or mutated Pc promoters carried on pPc2-1, pPc2-7 to pPc2-10 and pPc2-5 and pPc2-5^∗^; and Pc-*lacZ* transcriptional fusions with WT or mutated Pc2A and Pc2B carried on plasmids pPc2A, pPc2A^∗^, pPc2B and pPc2B^∗^, respectively. Inactivating mutation of a promoter -10 sequence is indicated by a cross. The wild type and mutated -10 sequences are written under the promoters with mutated residues shown in lowercases. Boundaries of the cloned fragment are indicated by black dots and numbered as in **Figure 1B**. The cloned portion of the *dfrA1* coding sequence is indicated by a rectangle. Broken arrows represent each Pc2 promoter, and their coordinates (1st base of its -35 element and last base of its -10 element) are indicated for individually cloned promoters. **(B)** The contribution of promoters Pc2A, Pc2B and Pc2C within *attI2* was estimated by measuring β-galactosidase activity from constructs carrying one or several inactivated promoters. **(C)** Individual promoter strength was estimated by measuring β-galactosidase activity. At least five independent assays were performed for each construct. Error bars indicate the standard deviation. *P*-values (ANOVA and HSD Tukey’s test) for comparisons with pPc2-1 **(B)** or with the respective WT promoter **(C)** are indicated: ^∗∗^*p* < 0.01, NS *p* > 0.05.

The coexistence of three potentially active Pc2 promoters (Pc2A–Pc2C) raised the question of their respective contributions to gene cassette expression. To address this question, we inactivated them individually or concomitantly in pPc2-1 (pPc2-7 to pPc2-10; **Figure 4A**), by placing mutations at key positions in their respective putative -10 elements (**Table 1**). As shown in **Figure 4B**, inactivation of Pc2C in pPc2-1 (pPc2-10) had no significant effect on β-galactosidase expression, though Pc2C inactivation in pPc2-5 abolished all activity (**Figure 4C**). In contrast, inactivation of Pc2A (pPc2-7) or Pc2B (pPc2-8) reduced the overall expression level by 53 and 32%, respectively (**Figure 4B**). Surprisingly, when both Pc2A and Pc2B (pPc2-9) were mutated, 25% of the initial pPc2-1 β-galactosidase activity remained (**Figure 4B**). Indeed, as Pc2C did not appear to contribute to the activity from pPc2-1, we expected that double mutation of Pc2A and Pc2B would lead to a complete loss of activity. However, as mutation of Pc2A reduced pPc2-1 activity by around 50%, a similar decrease should have been observed after Pc2B mutation, which was not the case (**Figure 4B**). We thus verified whether the mutation introduced in the putative -10 element of Pc2A and Pc2B (**Table 1**) resulted in complete abolition of promoter activity. We cloned the wildtype and mutated Pc2A and Pc2B promoter regions in fusion with *lacZ* (**Table 1**), and found that although the mutation in Pc2A abolished completely the promoter activity, the activity of mutated Pc2B (pPc2B^∗^) was reduced by only 60% compared to wildtype Pc2B (pPc2B) (**Figure 4C**), suggesting that its -10 sequence might differ slightly from that previously inferred (Genbank accession number AM261760). These results indicate that Pc2A and Pc2B contribute equally to gene cassette expression in class 2 integrons.

### Polymorphism of Class 2 Pc Promoters

There are several variants of the Pc promoter from class 1 and 3 integrons (Collis et al., 2002; Jové et al., 2010). We performed an *in silico* analysis of all class 2 integron sequences available online (May 2016), and found that six of the 220 analyzed sequences exhibited variations in Pc2A and Pc2B (accession numbers: DQ533990, DQ533991, EU780012, CP012363, CP012365, and KU736868). These six class 2 integrons all contain an A to G substitution converting the -10 element of Pc2A into TAAAGT, and a G to A substitution converting the -35 element of Pc2B into TTATAT. Hereafter, we will call these promoters Pc2A-V2 and Pc2B-V2, respectively. In one of these integrons, DQ533990, the -35 element of Pc2B-V2 is also duplicated.

**FIGURE 5:**
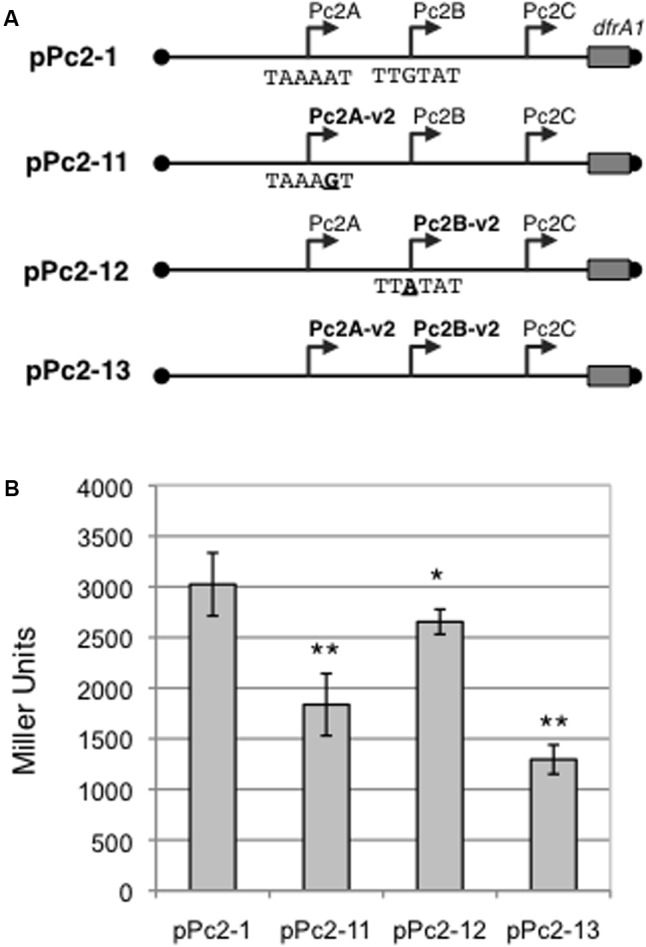
Strength of the Pc2A and Pc2B promoter variants. **(A)** Schematic representation of Pc-*lacZ* translational fusions with the entire *attI2* sites carrying combinations of promoters Pc2A, Pc2B, Pc2A-v2 and Pc2B-v2. Sequences of the -10 or -35 elements of, respectively, Pc2A and Pc2B promoters are written. Boundaries of the cloned fragment are indicated by black dots. The cloned portion of the *dfrA1* coding sequence is indicated by a rectangle. Broken arrows represent the Pc promoters. **(B)** The contribution of the Pc2A and Pc2B variants to gene cassette expression was estimated by measuring β-galactosidase activity from the various constructs. At least five independent assays were performed for each construct. Error bars indicate the standard deviation. *P*-values (ANOVA and HSD Tukey’s test) for the comparison with pPc2-1 are indicated: ^∗^*p* < 0.05, ^∗∗^*p* < 0.01.

To investigate the impact of this Pc2 polymorphism on the strength of promoters Pc2A/Pc2B, we introduced the Pc2A-V2 and/or Pc2B-V2 mutations in the pPc2-1 plasmid (**Figure 5A**). Replacing Pc2A by Pc2A-V2 or Pc2B by Pc2B-V2 significantly reduced *lacZ* expression by 39 and 12%, respectively (**Figure 5B**). When both promoters Pc2A and Pc2B were replaced by their variants Pc2A-V2 and Pc2B-V2, there was an additive effect, and β-galactosidase expression fell by 57% compared to that obtained with pPc2-1 (**Figure 5**).

Interestingly, the Pc2A-V2 and Pc2B-V2 variants were always found associated only in class 2 integrons encoding a complete integrase gene, with no premature STOP codon, that has been shown to encode a functional IntI2 protein (Hansson et al., 2002). None of these variants could be recovered in class 2 integrons encoding a truncated integrase.

## Discussion

This work highlights particularities in the expression of the integrase and gene cassettes of class 2 integrons. After mapping the P*intI2* promoter (**Figure 1**), we showed that despite the presence of a potential LexA box, LexA did not bind this region. Thus, integrase expression is not regulated by the SOS response in class 2 integrons, contrary to what has been shown for class 1 and *V. cholerae* integrons (Guerin et al., 2009). Interestingly, Cambray et al. (2011) found a correlation between the lack of a LexA binding box and an inactive integrase. In their analysis, four classes of integron, including class 2, did not fit this general scheme, as they displayed both a putative LexA operator and an inactive integrase (Cambray et al., 2011). As we show here that *intI2* expression is not SOS-dependent, class 2 integrons also comply with this general model, belonging to integron classes in which the absence of LexA control correlates with an inactive integrase. Closer examination of the class 2 integron putative LexA operator (CTGTATAGGCAGACAG) revealed the presence of 4 C/Gs, with a stretch of 3 consecutive C/Gs, in the 10-bp central variable region, whereas most experimentally validated LexA operators in *E. coli* display only 3 or fewer C/Gs in this region^1^. One can hypothesize that the lack of the usual TA stretch within the class 2 integron putative LexA site may explain why LexA does not bind this site. In the three other integron classes with both a putative LexA operator and an inactive integrase, the putative LexA operator includes 0 or 1 C/G in the central region (Cambray et al., 2011). In class 1 integrons, besides LexA-dependent regulation, expression of the integrase gene is also inhibited by transcriptional interference from the strong Pc variant, due to the face-to-face arrangement of the Pc and P*intI1* promoters (Guerin et al., 2009). In class 2 integrons, the Pc promoters (Pc2A and Pc2B) and P*intI2* are arranged tail-to-tail, so no such transcriptional interference can exist and *intI2* should be constitutively expressed. Consistently, we found no significant difference in β-galactosidase activity with pPintI2-2 (P*intI2* cloned alone) and pPintI2-1 (entire *attI2* site, includes the Pc2 promoters) (**Figure 2**). Although *intI2* is fully expressed, the encoded integrase is not functional, owing to the ochre codon in position 179. Hansson et al. (2002) suggested that this shortened 178-aa IntI2 peptide might interfere with the *attI2* site, preventing any IntI-mediated recombination. This hypothesis could explain the stability of the gene cassette array of class 2 integrons (Hansson et al., 2002). Consistently, the rare class 2 integrons that encode an active IntI2 integrase display a broader range of gene cassettes and gene cassette arrays (Supplementary Figure S1).

This work also highlights two specificities of gene cassette expression in class 2 integrons: (i) the Pc2 promoter is located in the *attI* region and not within the *intI* gene as in other integrons (Collis and Hall, 1995; Collis et al., 2002; Baharoglu et al., 2012), and (ii) at least two promoters, Pc2A and Pc2B, are involved in gene cassette expression. Although we observed weak activity of the putative Pc2C promoter when Pc2A and Pc2B were removed (**Figure 3B**), Pc2C inactivation within the entire *attI2* region had no effect on β-galactosidase activity (**Figure 4B**). This indicated that the contribution of Pc2C to gene cassette expression is negligible which is consistent with its structure, since it displays a suboptimal 19-bp long spacer between the -35 and -10 elements (Mitchell et al., 2003).

The permanent coexistence of two functional Pc promoters within the integron *attI* region is a unique feature of class 2 integrons. In class 1 integrons, two gene cassette promoters namely Pc and the P2 have been also described but in only 10% of the integrons (Collis and Hall, 1995; Jové et al., 2010). The biological reason for the presence of two active Pc promoters in class 2 integrons is unclear. Further studies are needed to determine whether the presence of these two functional gene cassette promoters in class 2 integrons might be linked to differential regulation of these promoters. One can hypothesize that this peculiar organization might be linked to specific activation of Pc2A and/or Pc2B, either under specific conditions, e.g., in response to distinct lifestyle conditions, or by specific partner proteins.

As in class 1 and 3 integrons (Collis and Hall, 1995; Correia et al., 2003; Jové et al., 2010), there are several Pc variants of different strengths in class 2 integrons. We show here that the class 2 integron promoters Pc2A-V2 and Pc2B-V2 variants are less efficient than Pc2A and Pc2B for gene cassette expression (**Figure 5**). Interestingly, the Pc2A-V2 and Pc2B-V2 variants have so far always been found together, in association with class 2 integrons that encode a functional IntI2 integrase (Barlow and Gobius, 2006; Márquez et al., 2008; Rodríguez-Minguela et al., 2009; Wei et al., 2014). This observed inverse correlation between the level of gene cassette expression and integrase activity raises another analogy with the class 1 integron model in which the weaker the Pc variant, the more efficient the IntI1 integrase (Jové et al., 2010).

Taken together, our data reveal the existence of two categories of class 2 integrons. The most prevalent category efficiently expresses a limited pool of gene cassettes from promoters Pc2A and Pc2B but is unable to modify its gene cassette array (non-functional IntI2). The rarer category produces an active integrase that permits gene cassette acquisition/rearrangement but expresses the gene cassettes more weakly (twofold; **Figure 5**). The rarity of functional class 2 integrons may be due to a high fitness cost associated with constitutive *intI2* expression. Indeed, even though constitutive *intI2* expression might represent an advantage because integrase production is not conditional on an environmental stimulus, it can also be a drawback, given the potential biological cost of *intI2* expression or activity. Indeed, it is known than in *Acinetobacter* and in *E. coli*, expression of the class 1 integron integrase gene is deleterious, with a high fitness cost, and this can lead to inactivation of the integrase (Starikova et al., 2012; Lacotte et al., 2017).

**FIGURE 6:**
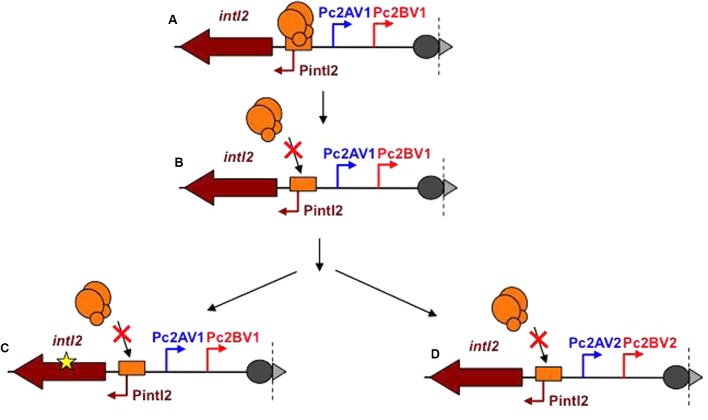
Model of evolution of *intI* and Pc promoters in class 2 integrons. **(A)** Ancestral class 2 integron. The *intI2* gene encodes a functional IntI2 integrase. LexA binds its operator and represses the expression of *intI2*. The Pc2 promoters are the V1 versions (high level of cassette gene expression). **(B)** Evolution of the LexA operator preventing LexA binding leading to derepression of *intI2* expression. Constitutive expression of *intI2* increases the fitness cost of the class 2 integron. This higher fitness cost is counterbalanced through mutations **(C)**, introducing of a premature STOP codon in the IntI2 coding sequence, releasing the fitness cost, and giving raise to the prevailing current class 2 integrons; **(D)** weakening the Pc2 promoters (Pc2AV2 and Pc2BV2) but keeping an active integrase, this evolution pathway being rare.

We propose an evolutionary model for class 2 integrons in which the expression of the ancestral class 2 integrons integrase was under control of the SOS response (**Figure 6A**). Then, mutation in the LexA operator would have led to a constitutive expression of the integrase gene encoding a fully active IntI2 (**Figure 6B**). The resulting high fitness cost would have lead to the introduction of either (i) a nonsense mutation in the *intI2* gene that inactivated IntI2 but maintained an array of highly expressed gene cassettes; this would have given rise to the currently prevailing class 2 integrons (**Figure 6C**), or (ii) mutations within the Pc promoters, generating variants of weaker activity, in order to reduce gene cassette expression and the potentially associated fitness cost (**Figure 6D**). This latter group of class 2 integrons constitutively express a functional IntI2 whose high fitness cost may explain their rarity.

## Author Contributions

M-CP and TJ conceived the study. M-CP coordinated the study. TJ, AT, and SDR performed the experiments. TJ, M-CP, and SDR analyzed the data and wrote the manuscript. AG-S revised the manuscript.

## Conflict of Interest Statement

The authors declare that the research was conducted in the absence of any commercial or financial relationships that could be construed as a potential conflict of interest.

## Footnotes

1 http://regulondb.ccg.unam.mx/
