# Corrigendum: Gene Expression in Class 2 Integrons Is SOS-Independent and Involves Two Pc Promoters

**DOI:** 10.3389/fmicb.2017.02378

**Published:** 2017-11-27

**Authors:** Thomas Jové, Sandra Da Re, Aurore Tabesse, Amy Gassama-Sow, Marie-Cécile Ploy

**Affiliations:** ^1^INSERM, CHU Limoges, UMR 1092, Université Limoges, Limoges, France; ^2^Unité de Bactériologie Expérimentale, Institut Pasteur de Dakar, Dakar, Senegal

**Keywords:** integrons, SOS response, promoter, antibiotic resistance, regulation

In the original article, the reference (Baharoglu et al., 2012) incorrectly replaced (Krin et al., 2014). It should be Krin, E., Cambray, G., and Mazel, D. (2014). The superintegron integrase and the cassette promoters are co-regulated in *Vibrio cholerae. PLoS ONE* 9:e91194.

The authors apologize for this error and state that this does not change the scientific conclusions of the article in any way.

## Conflict of interest statement

The authors declare that the research was conducted in the absence of any commercial or financial relationships that could be construed as a potential conflict of interest.
