# Supplementary material for: Gene Expression in Class 2 Integrons Is SOS-Independent and Involves Two Pc Promoters
Source: Front Microbiol. 2017 Aug 15;8:1499. doi: 10.3389/fmicb.2017.01499 (PMC5559693; doi:10.3389/fmicb.2017.01499)
Supplement: Supplementary file 1 [file Table_1.PDF]

Table S1: Primers used in this study.

Primers		Sequence (5’-3’)
**Number**	**Name**	
1	Pint2L	CCGGAATTCATTTTATTAGTAAGCATGCTC
2	Pint2R	GCGGGATCCATAAAACGCTCCTTGTCTTGCAAC
3	Pint2bL	CCGGAATTCGTAAACAAGCATCTCTAGGCG
4	PintI2mutL	TTAAATCCCAGCCGGACGAGGCTGTATAGGC
5	PintI2mutR	CTATACAGCCTCGTCCGGCTGGGATTTAAAAAGTG
6	LexAmutL	AGGCTGTATAGGCAGAACTTTGCAAGACAAGGAG
7	LexAmutR	CTTGTCTTGCAAAGTTCTGCCTATACAGCCTATTC
8	Int4b	CCGGAATTCACACCGTGGAAACGGATGAAG
9	DORF11	CGCGGATCCATCGTTGCTGCTCCATAACA
10	Pc2L	CCGAATTCTCGCATATCCGTGCGTATAGA
11	Pc2DR	GCGGGATCCATGGCTGTTTCCTTAAACAAGCATCTCTAGGCG
12	Pc2R	CGCGGATCCACTCCATTCTTCGATATAGCTAC
13	Pc2IntI2L	CCGGAATTCCAACGGCTGTTGTAAAAACC
14	Pc2AL	CCGGAATTCGTGCCTGTTTTTACGCCTA
15	Pc2BL	CCGGAATTCCAGGATAGACGGCATGCACG
16	Pc2CL	CCGGAATTCAGCGGGTGACAAAACGAG
17	PC2DFRL	CCGCCGCCGGAATTCTTAACCTCTGAGGAAGAATTGTG
18	Pc2AR	GCGGGATCCCATGGCTGTTTCCTATCTCACCCAAAGAACTC
19	Pc2BR	GCGGGATCCATGGCTGTTTCCTATGCTTACCGTTAATTAATATAATC
20	Pc2AmutL	GCTAAATAAAT**CG**A**GCG**GTTATGAGTTCTTTGGGTG
21	Pc2AmutR	AAGAACTCATAAC**CGC**T**CG**ATTTATTTAGCAT
22	Pc2BmutL	AAGAAAGTCTATT**CG**AT**G**CAAGTGATTATATTAATTAAC
23	Pc2BmutR	ATAATCACTTG**C**AT**CG**AATAGACTTTCTTTAAAAATAC
24	Pc2CmutL	ACGAGCATGCTTACTAA**CG**AAATGTTAACCTCTGAGG
25	Pc2CmutR	GAGGTTAACATTT**CG**TTAGTAAGCATGCTCGTTTTGTC
26	Pc2Amut2L	GCTAAATAAATTAAA**G**TGTTATGAGTTCTTTGGGTG
27	Pc2Amut2R	AAGAACTCATAACA**C**TTTAATTTATTTAGCAT
28	Pc2Bmut2L	GTAATAACAGAGTGTCTT**A**TATTTTTAAAGAAAG
29	Pc2Bmut2R	TTAAAAATA**T**AAGACACTCTGTTATTACAAATCGTG
