# Supplementary figures and images for: Gene Expression in Class 2 Integrons Is SOS-Independent and Involves Two Pc Promoters
Source: Front Microbiol. 2017 Aug 15;8:1499. doi: 10.3389/fmicb.2017.01499 (PMC5559693; doi:10.3389/fmicb.2017.01499)

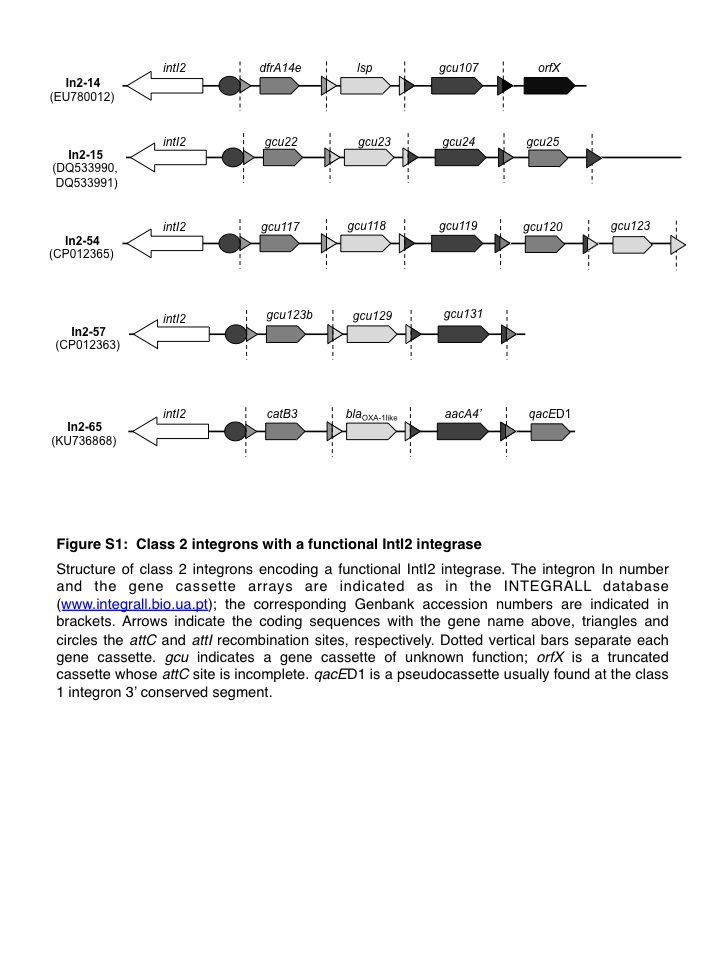

Supplement: Supplementary file 2 [file Image_1.tiff]
